# Fault Diagnosis of Rotating Machinery Based on an Adaptive Ensemble Empirical Mode Decomposition

**DOI:** 10.3390/s131216950

**Published:** 2013-12-09

**Authors:** Yaguo Lei, Naipeng Li, Jing Lin, Sizhe Wang

**Affiliations:** State Key Laboratory for Manufacturing Systems Engineering, Xi'an Jiaotong University, No. 28 Xianning West Road, Xi'an 710049, China; E-Mails: li3112001096@163.com (N.L.); jinglin@mail.xjtu.edu.cn (J.L.); wang563157979@stu.xjtu.edu.cn (S.W.)

**Keywords:** adaptive ensemble empirical mode decomposition, fault diagnosis, sifting number, added noise, rotating machinery

## Abstract

The vibration based signal processing technique is one of the principal tools for diagnosing faults of rotating machinery. Empirical mode decomposition (EMD), as a time-frequency analysis technique, has been widely used to process vibration signals of rotating machinery. But it has the shortcoming of mode mixing in decomposing signals. To overcome this shortcoming, ensemble empirical mode decomposition (EEMD) was proposed accordingly. EEMD is able to reduce the mode mixing to some extent. The performance of EEMD, however, depends on the parameters adopted in the EEMD algorithms. In most of the studies on EEMD, the parameters were selected artificially and subjectively. To solve the problem, a new adaptive ensemble empirical mode decomposition method is proposed in this paper. In the method, the sifting number is adaptively selected, and the amplitude of the added noise changes with the signal frequency components during the decomposition process. The simulation, the experimental and the application results demonstrate that the adaptive EEMD provides the improved results compared with the original EEMD in diagnosing rotating machinery.

## 1. Introduction

The signal processing technique based on vibration is one of the principal tools for diagnosing faults of rotating machinery [1–3]. It is possible to extract fault information from vibration signals by using the signal processing techniques. Empirical mode decomposition (EMD), as a time-frequency signal processing technique, has been developed to process nonlinear and non-stationary problems and widely applied to feature extraction and fault diagnosis of rotating machinery [4–7]. It is based on the local characteristic time scales of a signal and could decompose the complicated signal into a set of complete and almost orthogonal components named intrinsic mode function (IMF) [8,9]. The IMFs represent the natural oscillatory mode embedded in the signal and work as the basis functions, which are determined by the signal itself, rather than pre-determined kernels. EMD, however, has the problem of mode mixing, which is defined as either a single IMF consisting of components of widely disparate scales, or a component of a similar scale residing in different IMFs [10,11].

Consequently, ensemble empirical mode decomposition (EEMD), an improved version of EMD, was presented to solve the problem of mode mixing in EMD [10]. EEMD is a noise-assisted data analysis method. By adding finite white noise to the signal to be investigated, EEMD is supposed to eliminate the mode mixing problem. The performance of EEMD, however, depends on the parameters adopted in the EEMD algorithms, such as the sifting number, the amplitude of the added noise, *etc.* In most of the current studies on EEMD, these parameters were set as constant values. However, according to our investigation, different frequency components contained in signals have different sensitivities to these parameters [12]. As a result, the problem of mode mixing is not solved as expected and the performance of EEMD needs to be improved further.

Based on the investigation of the filtering behavior of EMD/EEMD and the relation between the signal frequency components and the amplitude of the added noise, we present a new adaptive ensemble empirical mode decomposition method in this paper. In this method, the sifting number is adaptively selected and the amplitude of the added noise varies with the signal frequency components during the decomposition process. By adopting both the adaptive sifting number and the adaptive added-noise amplitude, it is expected that the proposed EEMD method is able to improve the performance of the original EEMD in feature extraction and fault diagnosis.

The remainder of this paper is organized as follows. Section 2 briefly introduces the algorithm of EEMD. Section 3 is dedicated to a description of the proposed adaptive EEMD and generates a simulation to illustrate the method. In Section 4, experiments on a planetary gearbox test rig are conducted and vibration signals are collected to demonstrate the effectiveness of the proposed method in diagnosing gear faults. In Section 5, the proposed method is applied to diagnose an early fault of a heavy oil catalytic cracking machine set. The simulation, the experimental and the application results show that the adaptive EEMD produces the improved results compared with the original EEMD. Some concluding remarks are drawn in Section 6.

## 2. Ensemble Empirical Mode Decomposition

EEMD was developed by Wu and Huang to solve the problem of mode mixing of EMD [10]. It is a noise-assisted data analysis method, which defines the true IMF components as the mean of an ensemble of trials. Each trial contains the decomposition results of the signal plus a white noise of finite amplitude decomposed by EMD [10,11]. The principle of EEMD is as follows: the added white noise would populate the whole time-frequency space uniformly with the constituting components of different scales. Once a signal is added to this uniformly distributed white noise background, the components in different scales of the signal are automatically projected onto proper scales of reference established by the white noise in the background. Because each of the noise-added decompositions includes the signal and the added white noise, each individual trial may certainly generate a noisy result. But the noise in each trial is different in separate trials. Thus it can be decreased or even completely cancelled out in the ensemble mean of enough trails. The ensemble mean is treated as the true answer because finally, the only persistent component is the signal as more and more trials are added in the ensemble.

Based on the principle mentioned above, the EEMD algorithm can be given as follows [11].

(1) Initialize the number of ensemble *M*, the amplitude of the added white noise, and *m* = 1.
(2) Perform the *m*th trial on the signal added white noise.
  (a) Add a white noise series with the given amplitude to the investigated signal:
    (1) $$x_{m}(t)=x(t)+n_{m}(t)$$
    where *n_m_*(*t*) indicates the *m*th added white noise series, and *x_m_*(*t*) represents the noise-added signal of the *m*th trial.
  (b) Decompose the noise-added signal *x_m_*(*t*) into *I* IMFs *c_i_* (*i* = 1, 2, …, *I*) using EMD, where *c_i_*_,_*_m_* denotes the *i*th IMF of the *m*th trial, and *I* is the number of IMFs.
  (c) If *m* < *M* then go to step (a) with *m* = *m* + 1. Repeat steps (a) and (b) again and again, but with different white noise series each time.
(3) Calculate the ensemble mean *c_i_* of the *M* trials for each IMF.
  (2) $$c_{i}=\frac{1}{M}\sum_{m=1}^{M}c_{i,m},i=1,2,\ldots ,I,m=1,2,\ldots ,M\ldots$$
(4) Report the mean *c_i_* (*i* = 1, 2, …, *I*) of each of the *I* IMFs as the final IMFs.

**Figure 1.**
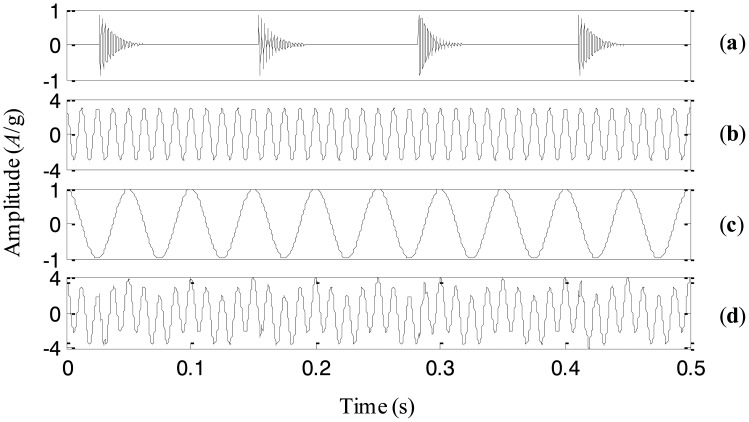
(**a**)–(**c**) the three components, and (**d**) the simulation signal.

EEMD is an improved version of EMD and is supposed to eliminate the problem of mode mixing by adding noise to the signal to change the distribution of extrema. The improvement of EEMD, however, largely depends on the parameters adopted in the EEMD algorithms, for example, the amplitude of the added noise. If the parameters vary, the decomposition results may change accordingly. To prove this statement, a simulation signal *x*(*t*) is considered here. It consists of three components: an impact component, a high-frequency sinusoidal wave and a low-frequency sinusoidal wave. The three components and the simulation signal are shown in Figure 1a–d, respectively.

**Figure 2.**
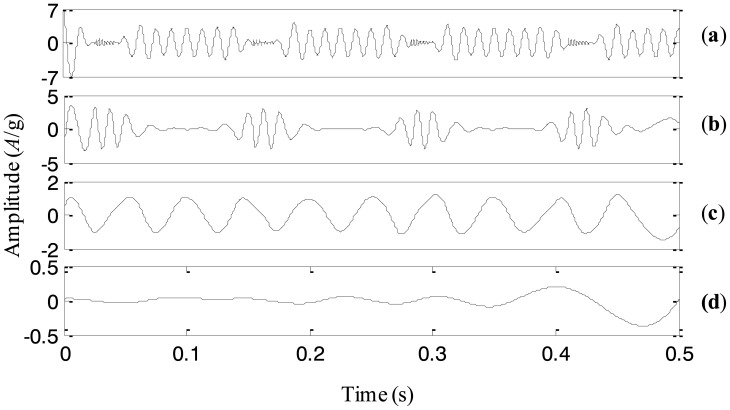
The decomposed result with the added noise amplitude of 0.001.

**Figure 3.**
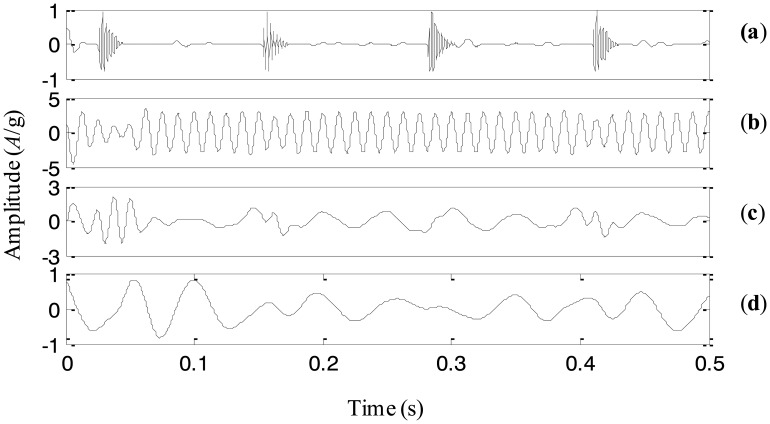
The decomposed result with the added noise amplitude of 0.01.

First, the signal is processed by EEMD with the added white noise amplitude of 0.001 of the standard deviation of the simulation signal. Correspondingly, four IMFs are generated and plotted in Figure 2a–d, respectively. It is obvious that the impact component and the high-frequency sinusoidal component are decomposed into the same IMF *c*_1_, *i.e.*, the mode mixing is occurring between higher frequency components. It could be explained that the added noise is too small to change the extrema distribution of the signal. Then, we process the simulation signal with the increased noise amplitude of 0.01. The decomposed IMFs are given in Figure 3a–d, respectively. The impact component and the high-frequency sinusoidal component are successfully decomposed into IMFs *c*_1_ and *c*_2_. However, the low-frequency sinusoidal wave is split into two IMFs *c*_3_ and *c*_4_. That is to say, the mode mixing appears in lower frequency components. It is probably because the added noise is too large and destroys the extrema distribution of lower frequency components, leading to the mode mixing.

Based on the simulation results, it is observed that in the process of EMD, high and low frequency components have different sensitivity to the intensity of the noise to be added in the investigated signal. The original EEMD method, however, adopts the constant noise amplitude and sifting number for all frequency components. Therefore, the problem of mode mixing is not overcome well and the performance of EEMD needs to be improved further.

## 3. The Proposed Adaptive Ensemble Empirical Mode Decomposition

### 3.1. The Proposed Method

**Figure 4.**
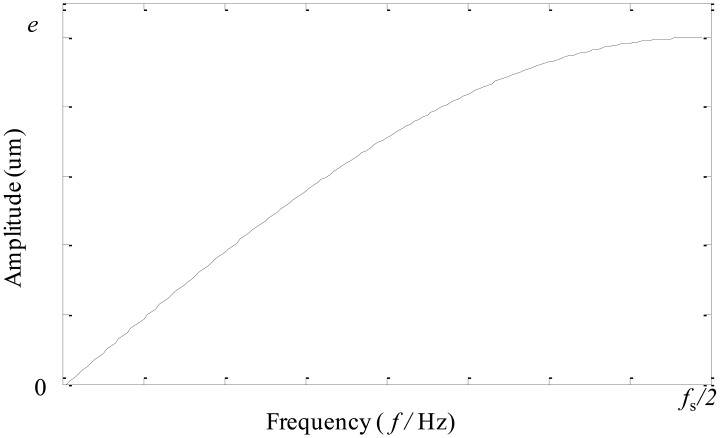
The spectrum of the noise constructed.

**Figure 5.**
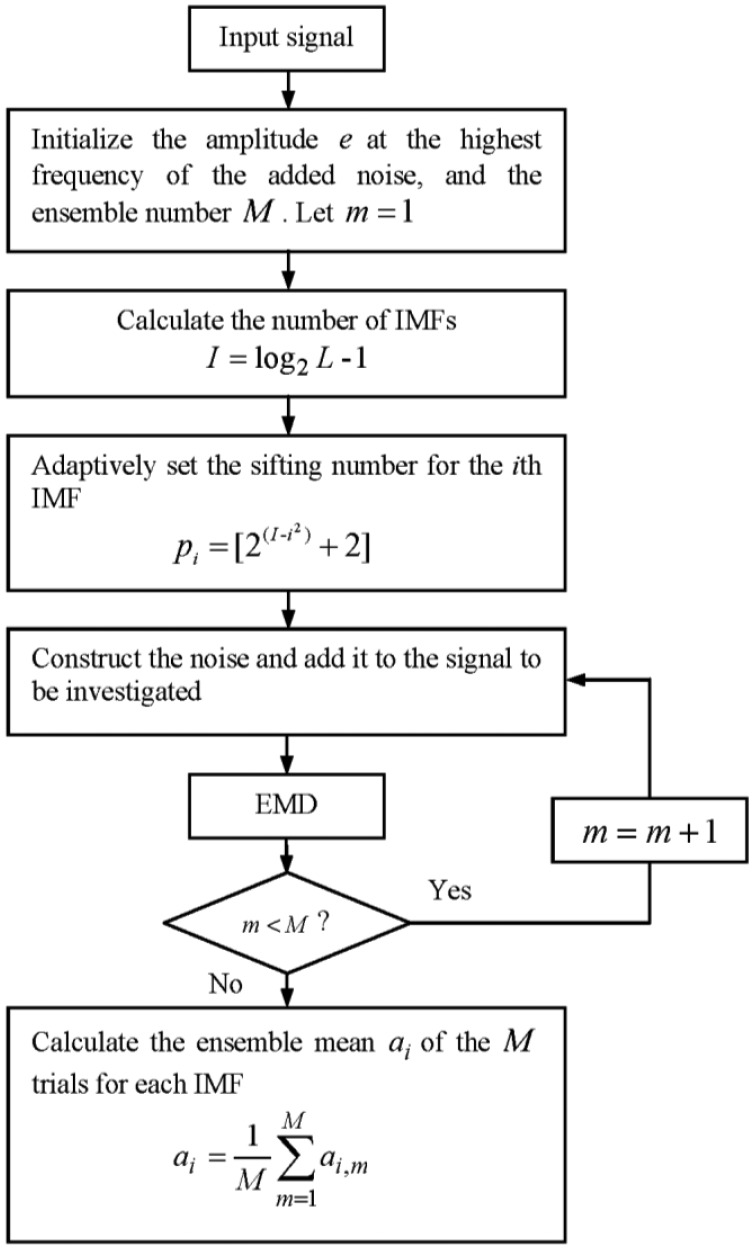
Flow chart of the adaptive EEMD.

In this section, an adaptive EEMD is proposed to further improve the original EEMD in solving the problem of mode mixing. In this method, according to different sensitivity of high and low frequency components to noise, larger noise and more sifting number are adopted in extracting high-frequency IMFs, while smaller noise and less sifting number are used in extracting low-frequency IMFs. To satisfy this requirement for noise, different kinds of noise are tried and tested. It is found that the noise having the amplitude changing as a sinusoidal function of the frequency performs best. Thus, the noise whose amplitude changes as a sinusoidal function of the frequency is constructed and utilized in the adaptive EEMD, instead of white noise adopted in the original EEMD. The frequency spectrum of the constructed noise is shown in Figure 4, in which *f*_s_ represents the sampling frequency and *e* denotes the amplitude at the highest frequency. The sifting number for each IMF is adaptively set following Equation (4). Figure 5 gives the flow chart of the adaptive EEMD algorithm. It includes the following procedural steps.

(1) Initialize the amplitude *e* of the highest frequency of the added noise, the number of ensemble *M*, generally *M* = 100 and *e* = 0.2. Let *m* = 1.
(2) Calculate the number of IMFs based on the signal length [10]:
  (3) $$I=\log_{2}L-1$$
  where *L* is the signal length.
(3) Adaptively set the sifting number *p_i_* for the *i*th IMF according to the following equation.
  (4) $$p_{i}=[2^{\left(I-i^{2}\right)}+2],i=1,2,\ldots ,I$$
  where [●] is a round operator.
(4) Construct the noise as shown in Figure 4 and add it to the signal to be investigated.
(5) Perform EMD on the added-noise signal and obtain the *m*th decomposition result *a_i_*_,_*_m_*.
(6) If *m* < *M* then go to step (4) with *m* = *m* + 1. Repeat steps (4) and (5).
(7) Calculate the ensemble mean *a_i_* of the *M* trials for each IMF and report the mean as the final IMF.
  (5) $$a_{i}=\frac{1}{M}\sum_{m=1}^{M}a_{i,m},i=1,2,\ldots ,I,m=1,2,\ldots ,M$$

### 3.2. Simulation Illustration

**Figure 6.**
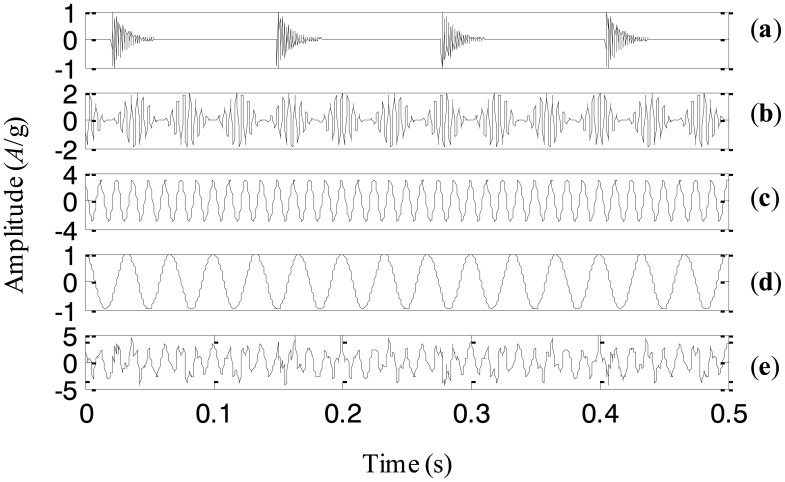
(**a**)–(**d**) the four components, and (**e**) the simulation signal.

A simulation signal is generated to illustrate the proposed adaptive EEMD method in this section. Since modulation and impact are two typical fault events in rotating machinery, the simulation signal includes modulation and impact components. It also consists of a high-frequency sinusoidal wave and a low-frequency sinusoidal wave respectively to represent certain rotating frequencies of machinery. Thus, there are four components corresponding to different physical meaning in the simulation signal. The four components and the simulation signal combined by them are shown in Figure 6a–e, respectively.

**Figure 7.**
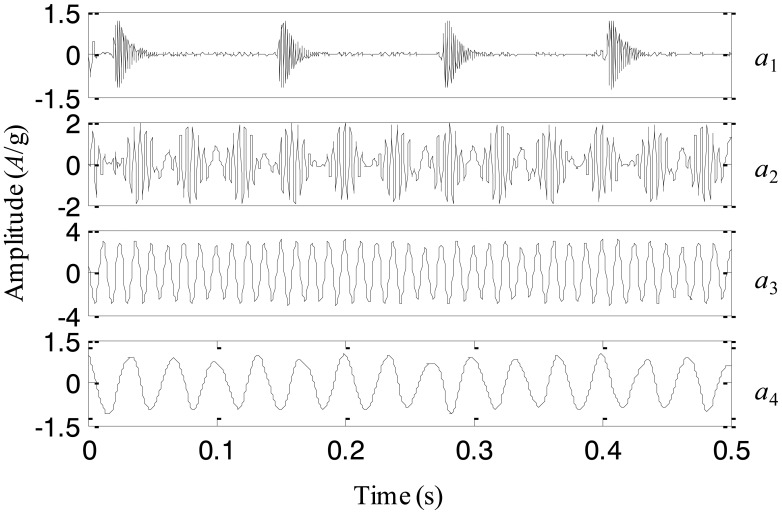
The IMFs using the adaptive EEMD for the simulation signal.

The adaptive EEMD method is utilized to process the simulation signal and the decomposed first four IMFs are plotted in Figure 7. It can be seen from the figure that IMFs 1–4 respectively correspond to the impact component, the modulation component, the high-frequency sinusoidal wave and the low-frequency sinusoidal wave. Comparing the decomposed IMFs in Figure 7 with the real components in Figure 6a–d, it is found that the different components embedded in the simulation signal are extracted accurately by the adaptive EEMD.

**Figure 8.**
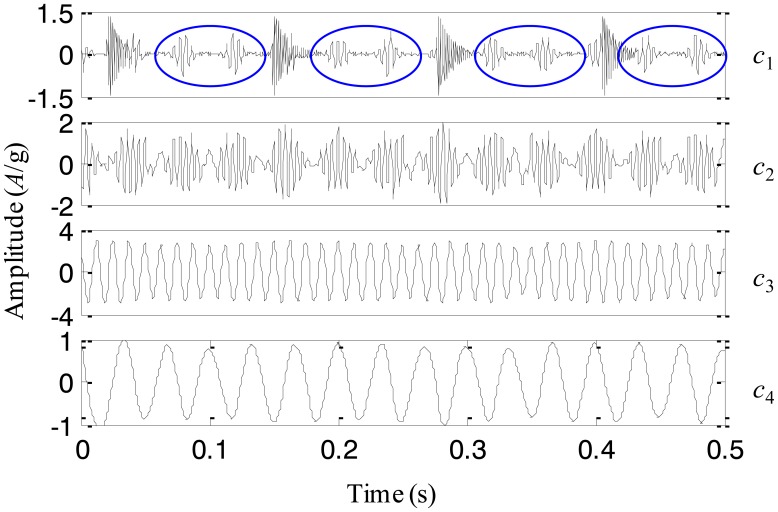
The IMFs using the original EEMD for the simulation signal.

For comparison, the simulation signal is analyzed using the original EEMD too and the amplitude of the added noise is 0.2 and the sifting number is 10. The decomposition result is displayed in Figure 8. It is seen that the problem of mode mixing appears between different IMFs and there are distortions for some IMFs. For example, the first IMF contains not only the impact component but also the modulation component. In addition, the amplitude of the second IMF corresponding to the modulation component changes obviously. This result implies that the original EEMD fails to produce the reasonable decomposition.

Based on the above simulation and comparison, it could be concluded that the adaptive EEMD is able to provide more accurate IMFs than the original EEMD, by adding noise having the amplitude varying as a sinusoidal function of the frequency into the signal, and adaptively changing the sifting number for different IMFs.

## 4. Experimental Demonstration for Fault Diagnosis of Planetary Gearboxes

Planetary gearboxes, as a kind of special gear transmission structures, are widely used in modern industry due to their advantages of large transmission ratio, strong load-bearing capacity, *etc.* They obviously differ from fixed-axis gearboxes and exhibit unique behaviors, which increase the difficulty of fault diagnosis [13–15].

**Figure 9.**
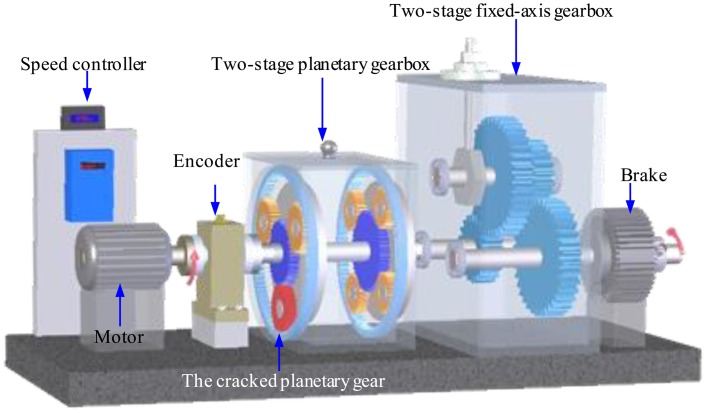
A schematic model of the planetary gearbox test rig.

**Figure 10.**
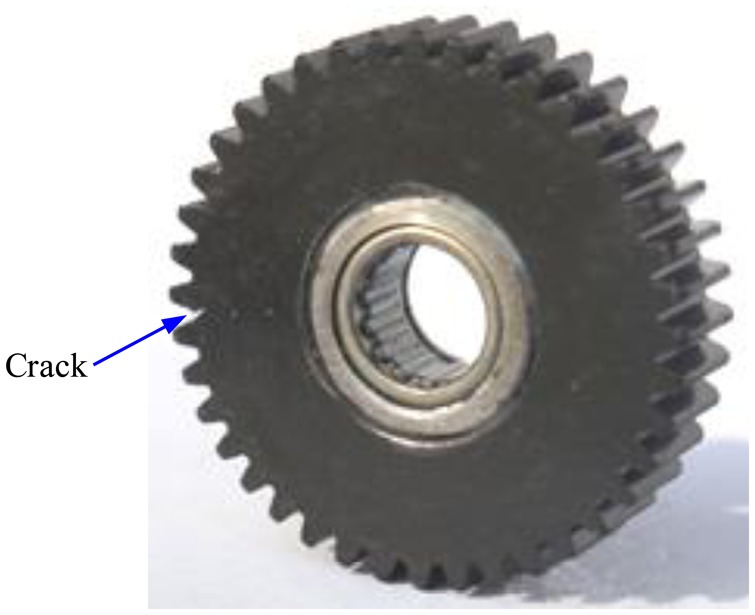
The cracked planetary gear.

In this section, experiments on a planetary gearbox test rig are conducted and vibration signals are captured to demonstrate the effectiveness of the adaptive EEMD in diagnosing gear faults. As given in Figure 9, the planetary gearbox test rig includes two gearboxes, a 3-hp motor for driving the gearboxes, and a magnetic brake for loading. One gearbox in the test rig is a planetary one and the other is a fixed-axis one. The planetary gearbox is our concern in this study, in which an inner sun gear is surrounded by several rotating planet gears, and a stationary outer ring gear [13]. A crack at the tooth root of one planetary gear is created in our experiments to simulate gear faults. The cracked planetary gear is shown in Figure 10.

An accelerometer is mounted on the planetary gearbox casing and is utilized to capture the vibration signals. The motor speed is about 20 Hz and the sampling frequency is set as 5,120 Hz. The experimental parameters and the characteristic frequencies of the planetary gearbox are shown in Table 1. It is noticed from the table that the rotating frequency of one planetary gear is 2.5 times as large as that of the carrier. Therefore, when the carrier rotates 2 cycles, the planetary gear meshes 5 periods with the ring gear, *i.e.*, 200 teeth. This tooth number is twice as large as that of the ring gear. That is to say, the ring gear meshes 2 periods with the planetary gear. In other words, the planetary gear returns to the initial position once the carrier rotates 2 cycles. For the carrier to finish rotating 2 cycles, it takes 2/3.33 = 0.6 s.

**Figure 11.**
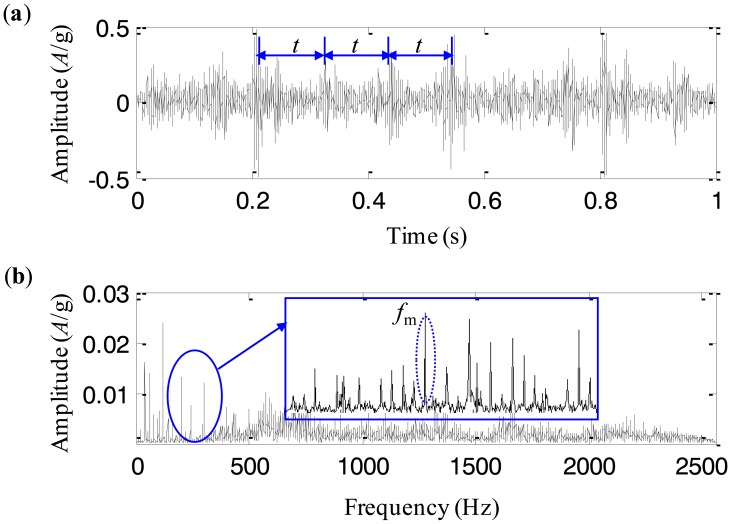
The experimental signal (**a**) time-domain waveform, and (**b**) frequency spectrum.

The collected vibration signal from the test rig with the cracked planetary gear is given in Figure 11a. Figure 11b displays its frequency spectrum. We notice that there are a series of impulses in the time-domain waveform. The period of the impulses is almost *t* = 0.1 s. It implies that the impulse frequency is 10 Hz. There are three planetary gears in the studied planetary gearbox, which pass the fixed accelerometer in turn. As a result, the pass frequency of the planetary gears equals 3 times as large as the rotating frequency of the carrier, *i.e.*, 10 Hz. It is obvious that the impulses in the time-domain waveform are caused by the rotation of the carrier, and therefore they are the vibration components of the normal gearbox. Besides these impulses, it is difficult to find any fault characteristics. The reason could be explained that the fault characteristics of the planetary gearbox are buried by the normal vibration components. The frequency spectrum of the vibration signal shown in Figure 11b is also analyzed and it is seen that there are rich sidebands around the mesh frequency. The interval of the sidebands is 3.33 Hz, which equals the rotating frequency of the carrier. Obviously, it is not the fault characteristics either. Thus, the fault characteristics of the cracked planetary gear are found from neither the time-domain waveform nor its frequency spectrum.

**Figure 12.**
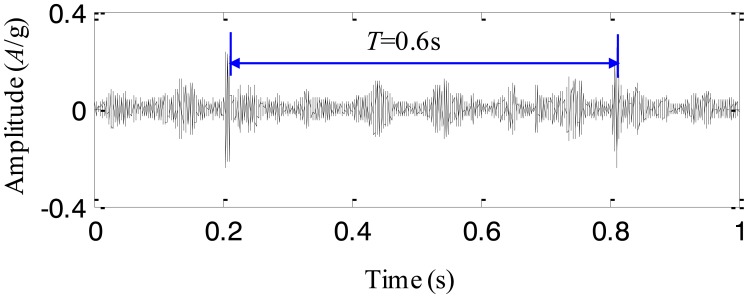
The first IMF extracted by the adaptive EEMD method.

**Figure 13.**
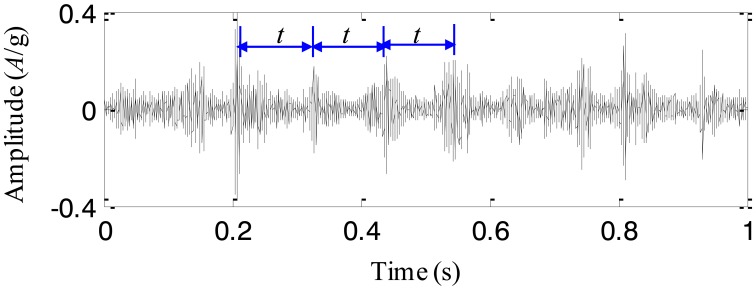
The first IMF extracted by the original EEMD method.

To extract the fault characteristics of the cracked planetary gear, the adaptive EEMD method is utilized to process the above signal. The first IMF extracted by the adaptive EEMD contains the richest information among all the IMFs and therefore it is selected for further analysis. The IMF is plotted in Figure 12. It is seen that there are impulses with the period *T* = 0.6 s. Based on the above analysis, it is concluded that once the carrier rotates 2 cycles, the cracked planetary gear returns to the initial position. Thus, the fault period of the cracked planetary gear is twice as large as the rotating period of the carrier, *i.e.*, 0.6 s. That is to say, the impulse component with the period *T* = 0.6 s is caused by the cracked planetary gear. Thus, the adaptive EEMD method is able to extract the fault characteristics from the normal components effectively. For comparison, the original EEMD with the sifting number 10 and the constant noise amplitude 0.2, is also used to process the same signal and the first IMF is given in Figure 13. Although it is observed that there are periodic impulses in the waveform of the IMF, the impulse (*T* = 0.6 s) caused by the cracked gear and those (*T* = 0.1 s) caused by the rotation of the carrier are decomposed in the same IMF, *i.e.*, the mode mixing happens. Through the comparisons, it is believed that the adaptive EEMD is more effective than the original EEMD in extracting fault characteristics of the planetary gearbox.

## 5. Application to Rub-Impact Fault Diagnosis of a Machine Set

**Figure 14.**
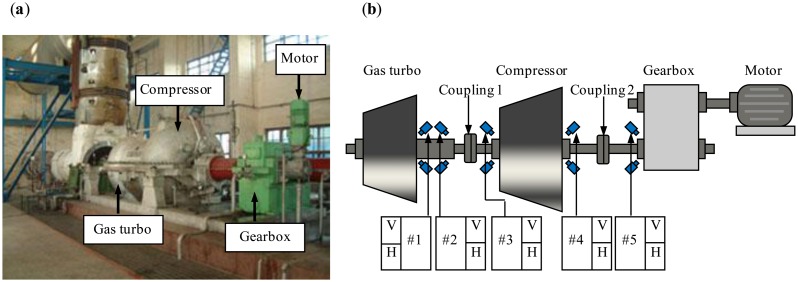
(**a**) One picture of the heavy oil catalytic cracking unit, and (**b**) structure sketch.

In this section, the adaptive EEMD method is applied to diagnosing an early rub-impact fault occurring in a machine set named heavy oil catalytic cracking unit in an oil refinery. A heavy oil catalytic cracking unit is one of the key machine sets and also typical rotating machinery used in oil refineries. It is significant to diagnose the faults of the heavy oil catalytic cracking unit as early as possible from the point of view of reducing loss. One picture of the machine set is given in Figure 14a. Figure 14b shows its structure sketch. This machine set includes a gas turbo, a compressor, a gearbox and a motor. Bushes #1 and #2 are to support the gas turbo shaft, bushes #3 and #4 are to support the compressor shaft, and bush #5 is to support the gearbox shaft. The hub and the laminas (left components of the gas turbo) on the shaft are a cantilever structure [16].

**Figure 15.**
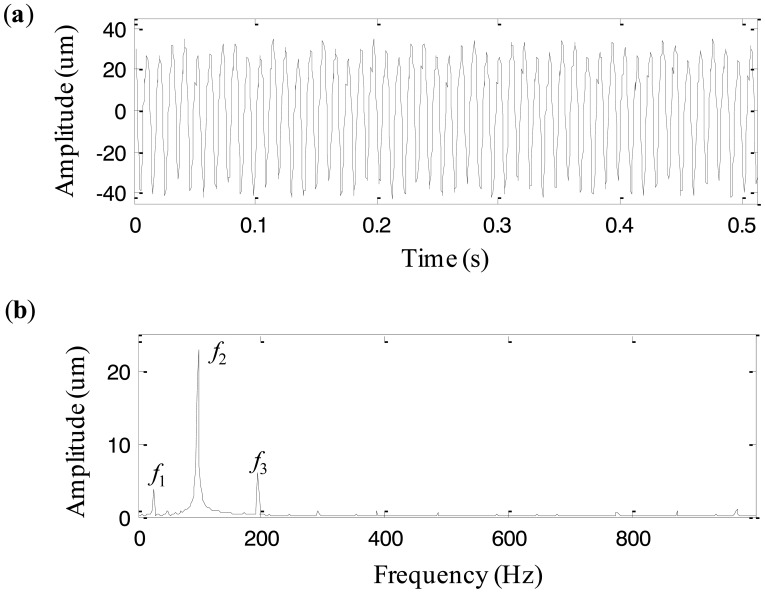
(**a**) The vibration signal at bush #5 of the machine set, and (**b**) the frequency spectrum.

The machine set generally operates under the rotating speed of 5859 rpm, *i.e.*, 97.65 Hz. Eddy current transducers are mounted on each bush case in vertical (V) and horizontal (H) directions respectively to acquire vibration signals with the sampling frequency of 2,000 Hz and the data length of 512. One day the operating condition of the machine set became abnormal and the abnormal vibration signals were recorded. Since the vibration at the measurement location of bush #5 was more intense than those of other locations, the vibration signal from this location is used to diagnose the fault. The signal is shown in Figure 15a. Figure 15b is its frequency spectrum, in which there are three dominant components and their frequencies are *f*_1_ = 25.35 Hz, *f*_2_ = 97.65 Hz, and *f*_3_ = 193.36 Hz, respectively. It is obvious that *f*_2_ is the rotating frequency, and *f*_3_ is a harmonic component, equaling two times rotating frequency of the machine set. *f*_1_ = 25.35 Hz, equal to 1/4 rotating frequency, seems to be a fractional harmonic of the rotating frequency. But it is verified by further investigation that it is the rotating frequency of the low-speed shaft of the gearbox. Therefore, we fail to find useful characteristics from the spectrum to diagnose the fault because the fault is still in its early stage and the characteristics are quite weak.

**Figure 16.**
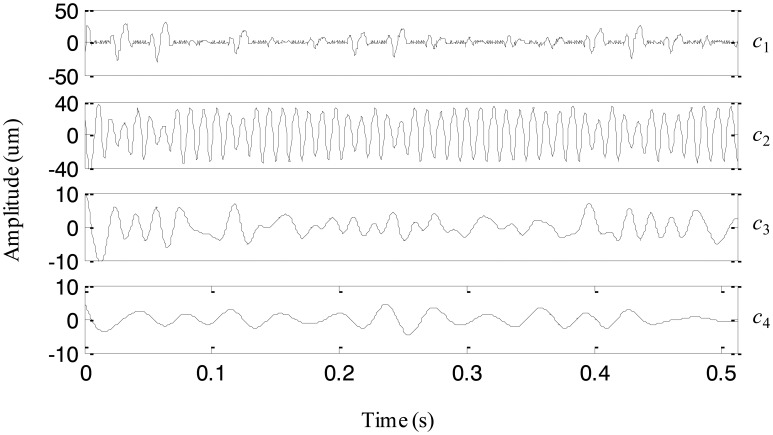
The IMFs extracted using the original EEMD for the vibration signal of the machine set.

To explore the cause leading to abnormality of the machine set, the original EEMD with the constant noise amplitude 0.2 and the fixed sifting number 10 is first used to analyze the signal in Figure 15a. The extracted four IMFs are plotted in Figure 16. From the first IMF, it seems that there are some small impulses, but they are not that obvious because the fault is still in its early stage. The second IMF represents the rotating frequency of the machine set. The third IMF is a periodic component with the 1/4 rotating frequency, which is the rotating frequency of the low-speed shaft of the gearbox. The forth IMF is generated due to the end effects and does not provide any useful information. Thus, it is difficult to judge the fault mode and cause based on the above IMFs extracted by the original EEMD.

**Figure 17.**
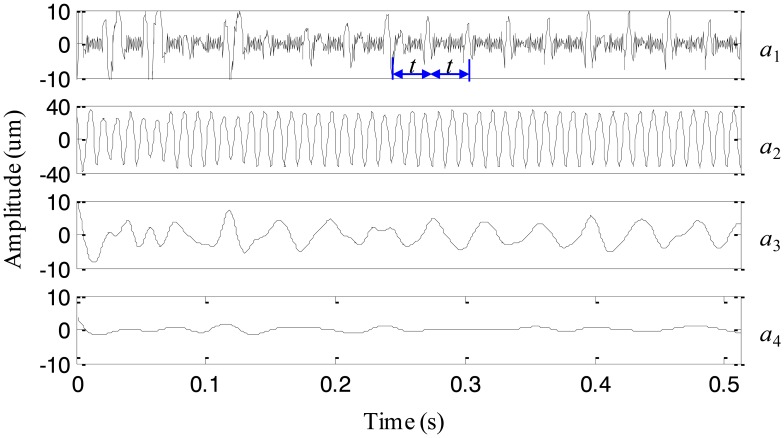
The IMFs extracted using the adaptive EEMD for the vibration signal of the machine set.

Then, the proposed adaptive EEMD is adopted to process the vibration signal. Figure 17 displays the decomposed four IMFs. A series of periodic impulses are seen in the first IMF and the interval between two adjacent impulses can be roughly estimated. It is about *T* = 0.031 s and approximately equals three times rotating period of the machine set, *i.e.*, 1/97.65 = 0.01024 s. It means that the impulse frequency is equal to 1/3 rotating frequency of the machine set. According to References [17,18], 1/3 fractional harmonic of the rotating frequency is a symptom of an early rub-impact fault in rotor systems. Therefore, it is concluded that there is an early rub-impact fault between the shaft and the bush in the machine set. In addition, IMFs 2–4 produced by the adaptive EEMD are similar to those decomposed by the original EEMD but much smoother than the latter. Actually, the rub-impact fault was confirmed by disassembling the machine set in the subsequent maintenance.

According to the comparisons between the adaptive EEMD and the original EEMD, it is verified that the adaptive EEMD is able to effectively extract the rub-impact features that cannot be detected from the vibration signal, the frequency spectrum, and the IMFs extracted by the original EEMD. Therefore the proposed method performs better in extracting early fault information.

## 6. Conclusions

This paper develops an adaptive ensemble empirical mode decomposition (EEMD) method to improve the original EEMD for fault diagnosis of rotating machinery. In the proposed method, the amplitude of the added noise varies with the signal frequency components and the sifting number is adaptively selected during the decomposition process. We use simulations to compare the adaptive EEMD and the original EEMD, and find that the former produces more accurate IMFs than the latter. The method is also demonstrated by detecting gear crack using the experimental data from a planetary gearbox test rig. Then it is applied to diagnosing an early fault occurring in a heavy oil catalytic cracking machine set. All results including simulations, experiments and applications reveal that the adaptive EEMD improves the performance of the original EEMD in feature extraction and fault diagnosis.

Although the proposed adaptive EEMD obtains improved decomposition over the original EEMD for the simulation, the experimental and the application of this study, we cannot guarantee that it works for all applications. The method is developed based on some experience and therefore it is not perfect enough. We are still thinking about how to improve the scientific soundness and the robustness of the method and hope that the improved result can be obtained and will be reported in near future.

## Figures and Tables

**Table 1.** Parameters and characteristic frequencies of the planetary gearbox.

**Tooth number of gears**			**Gear number**	**Rotating frequency (Hz)**			**Mesh frequency (Hz)**
Sun	Planetary	Ring	Planetary	Sun	Planetary	Carrier	--
20	40	100	3	20	8.33	3.33	333.33
